# Medical Papers in Public Libraries

**Published:** 1916-02-19

**Authors:** 


*To the Editor of* THE HOSPITAL.

### Medical Papers in Public Libraries.

*To the Editor of* THE HOSPITAL.

SIR,—Why should Worthing have all the praise? Your correspondent "B. R." declares that the Public Library of Worthing, by providing a weekly copy of THE HOSPITAL, is an exception to the rule by which such papers are usually excluded. Let "B. R." reassure himself (or herself). The practice of Worthing is also followed at Watford, where, Sir, I and my fellow-rate-payers can see the current issue of THE HOSPITAL every week. I venture to suggest that the only coincidence in this happy provision is that Worthing and Watford both begin with "W," and that the practice of the Watford and Worthing Public Libraries is followed in many other provincial towns which contain workers and readers engaged or interested in institutional life and affairs. The wonder at present would surely be that any public library did not contain "a newspaper dealing with medicine and its allied subjects"; but, if I may judge from my own experience, it is an interest in hospitals and also in medicine which brings readers to THE HOSPITAL's desk at the public library here.—Yours faithfully, A WATFORD OBSERVER.

February 12, 1916.

